# Accuracy and stability of accommodation and vergence responses during sustained near tasks in uncorrected hyperopes

**DOI:** 10.1038/s41598-023-41244-9

**Published:** 2023-09-01

**Authors:** Michael Ntodie, Kathryn Saunders, Julie-Anne Little

**Affiliations:** 1 Department of Optometry and Vision Science, School of Allied Health Sciences, College of Health and Allied Sciences, University of Cape Coast, Cape Coast, Ghana; 2 Centre for Optometry and Vision Science, Biomedical Sciences Research Institute, Ulster University, Coleraine, UK

**Keywords:** Refractive errors, Vision disorders

## Abstract

This study investigated the accuracy and stability of accommodative and vergence functions in children with and without hyperopia while engaged in two sustained near tasks. The sustained accommodative and vergence characteristics of participants without refractive correction (n = 92, aged 5–10 years) with and without hyperopia (defined as cycloplegic retinoscopy ≥  + 1.00D and less than + 5.00D) were measured using eccentric infrared photorefraction (PowerRef 3; PlusOptix, Germany). Binocular measures of accommodation and eye position were recorded while participants engaged in 2 tasks at 25 cm for 15 min each: (1) reading small print on an Amazon Kindle and (2) watching an animated movie on liquid crystal display screen. Comprehensive visual assessment, including measurement of presenting visual acuity, amplitude of accommodation, and stereoacuity was conducted. The magnitude of accommodative and vergence responses was not related to refractive error (*P* > 0.05). However, there were inter-task differences in the accuracy and stability of the accommodative responses across refractive groups (*P* < 0.05). The relationship between accommodation and vergence was not significant in both tasks (*P* > 0.05). However, increased accommodative and vergence instabilities were associated with total accommodative response (*P* < 0.05). Despite having greater accommodative demand, uncorrected hyperopes accommodate comparably to emmetropic controls. However, uncorrected hyperopes have increased instabilities in their accommodative and vergence responses, which may adversely impact their visual experience.

## Introduction

Hyperopia, a common refractive error in young children, presents a challenge to the clarity of near vision because of the additional accommodative burden exerted on the vision system which must compensate for the distance refractive error in addition to overcoming the dioptric demand of near targets^1,2^. Efficient near vision is increasingly more important for the social and educational well-being of children in general, and the hyperope in particular, as most tasks are performed at close/near working distances^3^. Compared to myopia, there are few studies which have investigated the accuracy and stability of the accommodative response in hyperopia. However, of the few studies reporting on hyperopes the consistent finding is that children with uncorrected hyperopia tend to demonstrate significant accommodative lags^1,4,5^. Furthermore, these studies report that children with higher magnitudes of hyperopia tend to demonstrate larger and more variable lags of accommodation^4^, and may be at risk for abnormal visual development including strabismus and amblyopia^2,4,6^. There is limited literature on the accommodative characteristics of the hyperopic child in relation to the sustained accommodative response, which reflects what happens naturally when, for example, the hyperopic child engages in near work at school for a prolonged period of time. Moreover, beyond conventional school tasks, children employ electronic devices such as smartphones and tablets for educational and recreational purposes for prolonged periods, suggesting that the accommodation and vergence systems will often be engaged for sustained periods. A single previous study by Roberts et al.^7^ explored the question of sustained accommodative accuracy and stability in children with uncorrected hyperopia, aged 3–10 years while engaged in two near activities. However, their study did not address the question of sustained vergence response in children with uncorrected hyperopia. A previous study which investigated the dynamic relationship between vergence and accommodation at a single time point, found that hyperopes simultaneously demonstrated accurate vergence responses alongside significant accommodative lag^1^. No previously published study has concurrently investigated the characteristics of young uncorrected hyperopes’ accommodation and vergence responses, in terms of accuracy and stability, and the interaction between the two systems during sustained near vision tasks. The purpose of this study was to investigate differences between the accuracy and stability of the accommodative and vergence responses in uncorrected hyperopes and emmetropic children while participants were engaged in two sustained typical daily near tasks with different visual demands; reading and watching a movie. Our hypothesis was that uncorrected hyperopes would have poorer accommodative and vergence responses than emmetropes and that there would be a difference in performance across tasks, with the movie task being less visually demanding^8^. We also hypothesized that hyperopes would fatigue more in their accommodative and vergences responses while performing these two sustained tasks.

## Materials and methods

### Study participants

Study participants included children aged 5–10 years (n = 92) who were recruited from a local Primary school, a community optometric practice and the Ulster University Optometry clinic, in Coleraine, UK^9^. The study adhered to the tenets of the Declaration of Helsinki and commenced after approval by the Ulster University Research Ethics Committee. For each participant, informed consent was obtained from their parents, and a short medical and ocular history obtained via a parental-administered questionnaire^9^. For the hyperopic group, inclusion criteria were participants with uncorrected hyperopia (n = 58; defined as participants without current or previous history of spectacle wear) whose spherical equivalent refraction in the least plus eye after cycloplegic retinoscopy was ≥  + 1.00D and less than + 5.00D, and with anisometropia less than 1.00D, and astigmatism less than 2.00DC^9^. Inclusion criteria for control group was emmetropia (n = 34) defined as a spherical equivalent refraction of − 0.25D to less than + 1.00D. For both groups, refractive error was defined by a cycloplegic retinoscopy result obtained using 1% drop Cyclopentolate hydrochloride. The refractive assessment was carried out after baseline visual assessments and the two sustained near tasks had been performed on a different day 1–2 weeks later. Comprehensive visual assessment including measures of presenting binocular distance and near visual acuity (Sonksen crowded LogMAR letter test at 3 m and 40 cm for distance and near respectively), stereoacuity (Frisby stereotest), ocular posture (prism cover test), near point of convergence (measured with the RAF rule^10^), accommodation amplitude (push-up test, measured with the RAF as previously described elsewhere^11,12^) and accommodative response (modified Nott dynamic retinoscopy) were obtained from all participants^9^. As all participants were non-spectacle wearers, all visual measures were taken without correction. Amplitude of accommodation was calculated from the accommodation amplitude determined by the push-up test plus the underlying spherical equivalent refractive error from the least plus eye. Exclusion criteria were: habitual spectacle wearers, presence of strabismus, a history of patching/penalisation amblyopia therapy and/or hospital eye service attendance, distance visual acuity worse than 0.2 LogMAR^13,14^ and those with refractive errors beyond the parameters described above.

### Sustained near tasks

Details of the two tasks, including testing time, distance and order of introducing tasks have been described in detail by the authors elsewhere^9^. Briefly, participants undertook two tasks, both of which were performed for a period of 15 min each including reading text and watching a movie on screens placed in downgaze at 25 cm. The choice of the reading and movie tasks enabled investigation of the characteristics of the accommodative and vergence responses under two different visual task demands, with the reading task constituting an “active/high” demand task, and the movie task a “passive/low” demand task^7,8^. In the reading (active) task designed to simulate a typical visually demanding activity undertaken at school, participants read aloud selected age-appropriate text (text size corresponded to a visual angle of 0.25 degrees, approximately 0.3LogMAR) on an Amazon Kindle^9^. The task was designed to be visually demanding to stimulate accommodation rather than as a test of reading ability (i.e. it was not challenging or complex text for the age of the child). Participants read from an Amazon Kindle presented at a near distance of 25 cm while simultaneous measurement of accommodation, gaze position (vergence), and pupil sizes were recorded by the PowerRef 3™ photorefraction system^9^. The Kindle, with a viewing window of 14° by 10.20° at 25 cm was housed in a wooden box with a forehead rest. Two Velcro straps around the head of the participant helped to minimise head movements during the task^9^. Prior to testing, participants were evaluated to determine their reading ability in relation to the choice of age-appropriate reading text, and to confirm that all participants could read the font size on the Kindle or customised reading material. For the youngest children (n = 26 aged 5–6 years; comprising of 19 hyperopes, and 7 emmetropes) who could not fluently read the simplest reading text on the Kindle, custom-made reading material was designed and presented on an LCD monitor of similar dimension to the Kindle^9^. The background illumination for the Kindle and monitor was 40 cd/m^2^ and 50 cd/m^2^ respectively (measured with ColorCal MK II™ Colorimeter), consistent with conventional usage of these devices^9^. This illumination setting provided sufficient contrast for the Kindle and monitor while allowing pupil sizes to be maintained within the operational range of the PowerRef 3™^15^.

In the movie (passive) task, participants watched an animated movie while simultaneous measurement of accommodation, vergence and pupillary response were recorded by the PowerRef 3™ at 25 cm^9^. The task was modelled after a recreational activity frequently undertaken by children. The target for the movie task was a popular, commercially available stop-motion animated movie containing broadband spatial frequency content^9,16,17^, chosen to engage and sustain interest and attention of participants. The movie target and the reading task were housed in the same set-up. Background illumination for the movie target ranged from range: 10–50 cd/m^2^ (mean 30 cd/m^2^)^9^.

### Set-up for the measurement of sustained accommodation

Measurement of sustained accommodation, gaze position (vergence), and pupil size were made with the PowerRef 3™ (PlusOptix, Germany) at a sampling frequency of 50 Hz. The photorefraction principle, from which the PowerRef 3 operates, has been utilised in many studies and described in detail previously^18,19^ and a thorough description of the set-up for the measurement of sustained accommodation and vergence in this study has been described by the authors elsewhere^9^. Briefly, the set-up consisted of the PowerRef 3™ camera, mounted on a custom-designed bench at 1 m ± 0.05 m. Two Periscopic mirrors were used to reflect infrared light from the instrument’s camera aperture into the eye^9,17^. The entire table was tilted by 16.7 degrees to enable participants to view in downgaze, thus adopting a more natural reading/viewing position. A lens calibration factor was obtained using a range of lenses (+ 4, + 3, + 2, + 1, − 2 & − 4 D), and incorporated into individual refraction estimates to increase precision of results^9,17,20^. For the few participants in whom individual calibration was unsuccessful, the group average calibration factor was applied^17^. Prior to participants undertaking the sustained tasks at 25 cm, a baseline measure of refractive state, eye position and pupil size was obtained at 1 m while participants viewed a Maltese cross target. The accommodative response during the two sustained tasks was computed as the difference in refraction at baseline (at 1 m, the manufacturer’s default instrument calibration for refractive error) and the 25 cm target demand as recorded by the PowerRef 3™^9^. The accuracy of this accommodative response was computed as the difference between the known dioptric demand of 4 D for the 25 cm target and the individual accommodative response measured with photorefraction^9,21,22^. For example, an emmetropic participant would yield a value of 0.00D refraction at baseline, and then, if they exhibited a typical accommodative response to the 4D target demand, they would measure a − 3.00D refraction for viewing distance of 25 cm, indicating that they produced a 3.00D response. The total accommodative response was calculated as the sum of the mean accommodative response to the target at 25 cm, and the accommodation needed to correct the subject’s underlying refractive error (equivalent to the participant’s cycloplegic refraction value measured on a different day). The vergence response was computed as the difference in gaze position between the right and left eyes (as recorded by the PowerRef 3™). The magnitude of vergence response to the target at 25 cm was calculated in metre angles as the difference in vergence at baseline (1 m) and the 25 cm target demand.

### Data analysis

**Figure 1:**
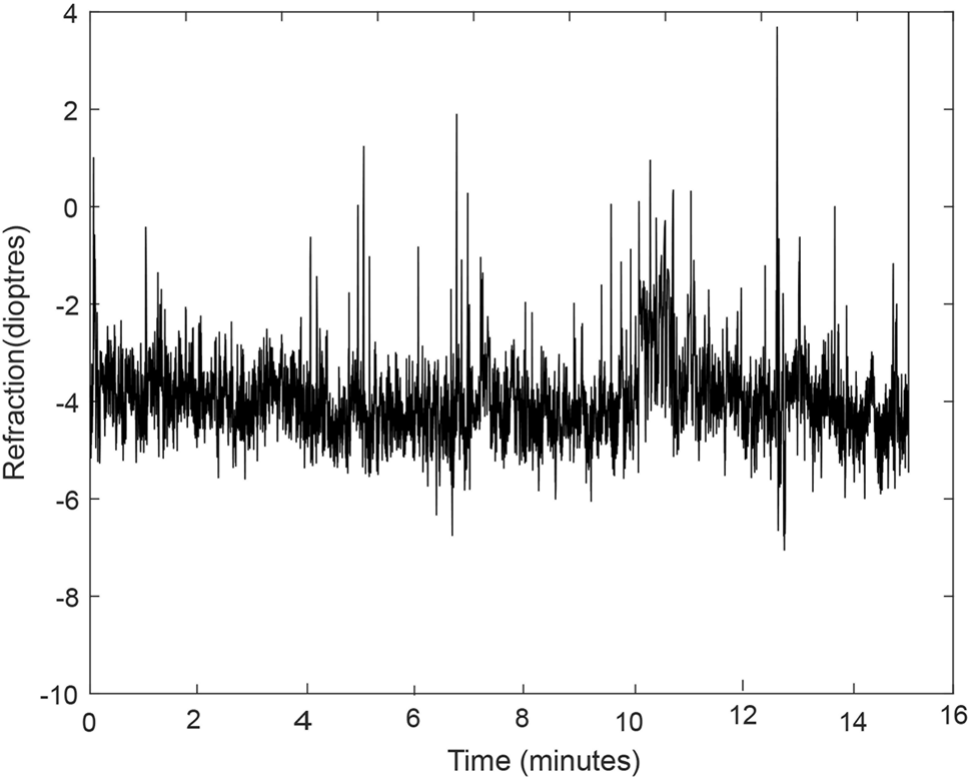
Raw traces of accommodative response over time in the movie task of participant in the study. The Y-axis represents refraction (from which the accommodative response was computed), and the X-axis represents time (duration) of the movie task.

Prior to undertaking statistical analyses, data samples from the PowerRef 3 were carefully inspected and processed in Matlab. Details of the data processing protocol used in the present study have been previously described by the authors^9^. For participants who read on the Kindle, data below the 5th percentile and above the 95th percentile in the vertical range were excluded to eliminate data arising from when the participants were reading the top and bottom of the page of text, as this up- and down-gaze could contaminate measurements. These outliers were therefore removed using the winsorization technique^23^. The outliers which were seen while participants read on the Kindle were non-existent in the movie task (Fig. 1).

The accuracy and stability of the accommodative response, as well as magnitude of the vergence response and its stability were analysed. The characteristics of the accommodative and vergence responses were analysed using the mean of all sampled data points across the 15-min time period.

The stability of the accommodative response was analysed in the time domain using the root mean square deviation of accommodative microfluctuations (RMS)^24,25^, while in the frequency domain, the Low Frequency Component (LFC < 0.6 Hz) of the response was obtained using the Fast Fourier Transform (FFT) in MATLAB^7,26,27^. The stability of the vergence response was investigated using the standard deviation of the mean vergence response across the 15-min time period. While vergence was calculated using eye position data from the two eyes, data from the eye with the least plus spherical equivalent refraction were used for accommodative analyses.

A two-factor repeated measure ANOVA was used to assess inter-task difference in the accuracy and stability of the accommodative and vergences responses. Pearson’s correlation (Bonferroni correction applied in multiple comparisons) was used to assess relationships between the accommodative and vergence responses and the magnitude of refractive error. Multivariate regression analysis was used to assess the relationship between the stability of the accommodative response and total accommodative response after adjusting for other variables. Statistical significance was set at* P* < 0.05.

## Results

The mean age of participants was 7.92 ± 1.54 years. All participants engaged in the two sustained near tasks. Other descriptive statistics of refractive error, accommodation, pupil sizes and baseline measures are presented in Table 1.

**Table 1:** Descriptive statistics of baseline visual measures and sustained accommodative and vergence functions in hyperopia and emmetropia. Spherical equivalent refraction was obtained from cycloplegic retinoscopy. Target distance for sustained tasks, and accommodative lag by Nott technique was at 25 cm (4 D).

Baseline measures	Refractive Group			Difference between refractive groups t-value, *P*-value
	All participants (n = 92) (Mean ± SD), range	Hyperopia (n = 58) (Mean ± SD), range	Emmetropia (n = 34) (Mean ± SD), range	
Age (years)	7.92 ± 1.54, (5, 10)	7.76 ± 1.62, (5, 10)	8.21 ± 1.37, (5, 10)	t = − 1.36, *P* = 0.18
Amplitude of Accommodation (D)	15.36 ± 3.16, (5.75, 22)	15.47 ± 3.32, (7, 22)	15.19 ± 2.93, (5.75, 20.25)	t = 0.40, *P* = 0.69
Near Point of Convergence (cm)	6.30 ± 1.67, (5, 15)	6.27 ± 1.18, (5, 12)	6.35 ± 2.28, (5, 15)	t = − 0.23, *P* = 0.82
Accommodative Lag (by Nott technique) (D)	0.37 ± 0.51, (− 1, 2.19)	0.33 ± 0.55, (− 1, 2.19)	0.44 ± 0.42, (− 0.35, 1.5)	t = − 0.97, *P* = 0.34
Spherical Equivalent refraction (D)	1.29 ± 0.88, (0, 4.38)	1.77 ± 0.73, (1, 4.38)	0.47 ± 0.30, (0, 0.83)	–
Sustained measures				
Accommodative Response (D) (reading task)	2.96 ± 0.78, (1.17, 5.46)	3.02 ± 0.84, (1.17, 5.46)	2.87 ± 0.67, (1.67, 4.38)	t = 0.89, *P* = 0.38
Accommodative Response (D) (Movie task)	2.28 ± 0.73, (0.47, 4.51)	2.27 ± 0.72, (0.47, 4.51)	2.29 ± 0.74, (1.12,4.35)	t = − 0.16, *P* = 0.87
Total Accommodative Response (D) (Reading task)	4.25 ± 1.24, (2.22, 7.96)	4.79 ± 1.18, (2.67, 7.96)	3.34 ± 0.67, (2.22, 4.88)	t = 6.59, ***P* < 0.0001**
Total Accommodative Response (D) (Movie task)	3.53 ± 1.14, (0.73, 6.91)	4.03 ± 1.02, (1.97, 6.91)	2.69 ± 0.78, (0.73, 4.35)	t = 6.57, ***P* < 0.0001**
Accommodative Stability [time-domain (D)] (Reading task)	0.13 ± 0.06, (0.03, 0.50)	0.14 ± 0.07, (0.03, 0.50)	0.12 ± 0.04, (0.06, 0.26)	t = 1.15, *P* = 0.26
Accommodative Stability [time-domain (D)] (Movie task)	0.15 ± 0.05, (0.07, 0.32)	0.15 ± 0.05, (0.07, 0.32)	0.14 ± 0.04, (0.10, 0.22)	t = 0.22, *P* = 0.83
Accommodative Stability [frequency-domain (Hz)] (Reading task)	7.65E−4 ± 7.42E−4 (1.54E−4, 4.86E−3)	8.26E−4 ± 8.34E−4 (1.54E−4, 4.86E−3)	6.62E−4 ± 5.49E−4 (2.12E−4, 3.19E−3)	t = 1.02, *P* = 0.31
Accommodative Stability [frequency-domain (Hz)] (Movie task)	1.20E−3 ± 7.71E−4 (2.22E−4, 4.55E−3)	1.21E-3 ± 7.36E-4 (2.22E-4, 4.55E-3)	1.19E-3 ± 8.39E-4 (2.26E-4, 3.34E-3)	t = 0.11, *P* = 0.92
Vergence Response (MA) (Reading task)	2.91 ± 0.49, (1.52, 4.10)	2.95 ± 0.47, (2.09, 4.10)	2.86 ± 0.52, (1.52, 4.06)	t = 0.84, *P* = 0.40
Vergence Response (MA) (Movie task)	2.67 ± 0.72, (0.44, 4.39)	2.65 ± 0.77, (0.44, 4.34)	2.70 ± 0.63, (0.85, 4.39)	t = -0.31, *P* = 0.76
Stability of Vergence Response (MA) (Reading task)	1.91 ± 0.19, (1.37, 2.37)	1.93 ± 0.17, (1.47, 2.34)	1.88 ± 0.22, (1.37, 2.37)	t = 1.12, *P* = 0.27
Stability of Vergence Response (MA) (Movie task)	1.34 ± 0.17, (0.87, 1.73)	1.36 ± 0.17, (0.98, 1.69)	1.31 ± 0.17, (0.87, 1.73)	t = 1.61, *P* = 0.11
Pupil size (mm) Reading task	5.11 ± 0.64, (3.75, 6.67)	5.12 ± 0.67, (3.75, 6.67)	5.10 ± 0.60, (3.96, 6.18)	t = 0.17, P = 0.87
Pupil size (mm) Movie task	5.36 ± 0.57, (4.04, 6.41)	5.42 ± 0.55, (4.05, 6.40)	5.24 ± 0.59, (4.04, 6.41)	t = 1.48 *P* = 0.14

MA—meter angle, t-test conducted was the two-sample t-test. Negative vergence value represents divergence. Accommodative & vergence target at 25 cm (4D stimulus).

Significant values are in bold.

### Relationship between accuracy of accommodation, type of task, refractive error, and total accommodative response

The accuracy of the mean accommodative response differed by task, with significant accommodative lag observed in the movie task (1.75 ± 0.76D) compared to the reading task (1.04 ± 0.78D), (F_(1,179)_ = 33.28,* P* < 0.0001; 2 × 2 mixed ANOVA analysis, dependent variable being mean accommodative lag, and factors being task type (reading/movie) and refractive group (emmetropes/hyperopes)). There was no group difference in results (F _(1, 179)_ = 0.52, *P* = 0.71), and no significant interactions observed between the two factors (F _(1, 179)_ = 0.39, *P* = 0.54).

**Figure 2:**
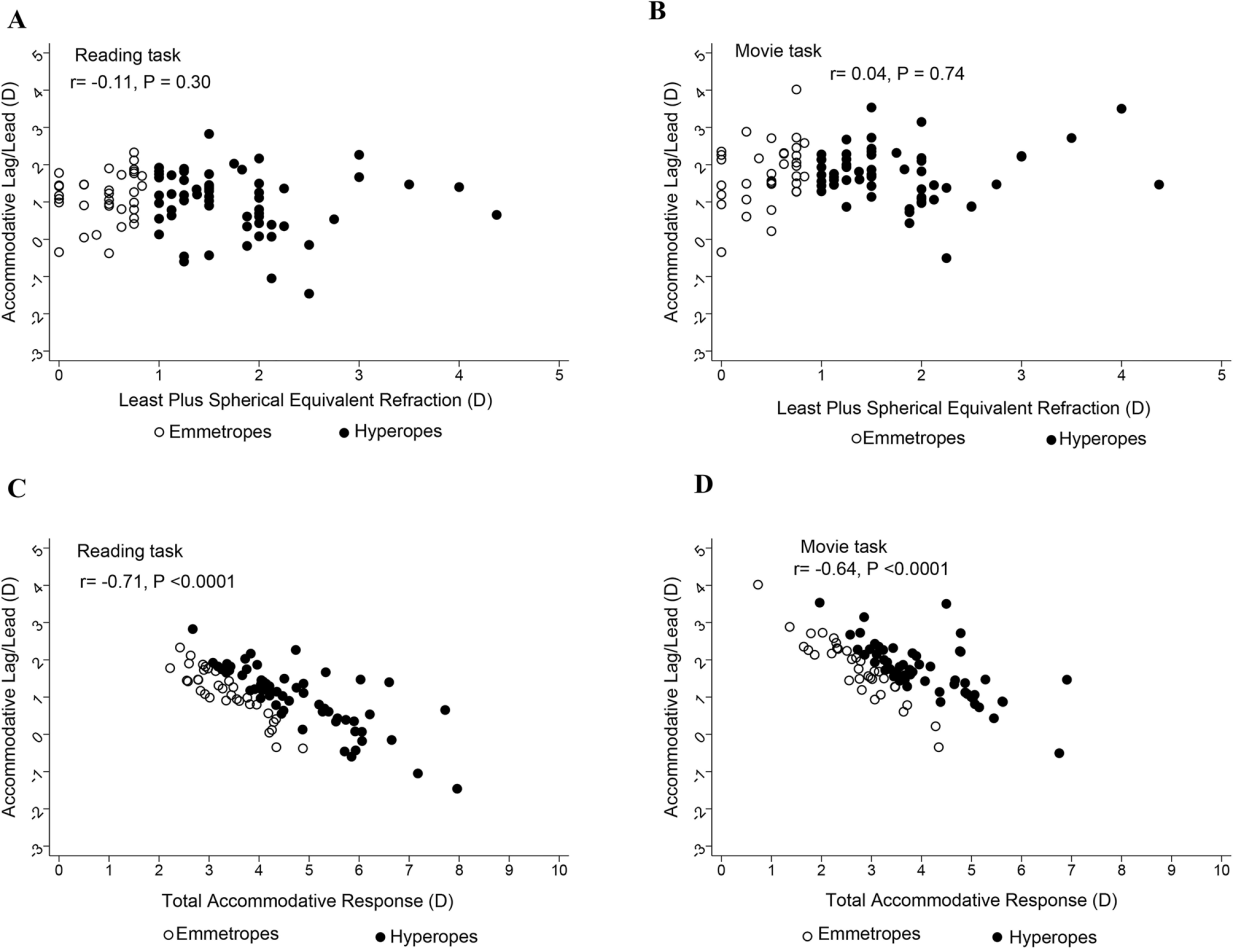
Scatter plots showing relationship between accuracy of accommodative response (lag/lead) and refractive error in the reading (**A**) and movie (**B**) tasks in all participants. Individual data points above zero indicate lag of accommodation, and those below indicate lead of accommodation. The relationship between the accuracy of the accommodative response and the total amount of accommodation in the reading and movie tasks are shown in panels (**C**) and (**D**). Black hollow circles represent participants with emmetropia, while black solid circles represent participants with hyperopia.

The relationship between accommodative accuracy (lag/lead) and refractive error (least spherical equivalent refraction) across the two tasks was not statistically significant in the two groups (r = − 0.11, *P* = 0.30; and r = 0.04, P = 0.74 for reading and movie tasks respectively, (Fig. 2A,B).

A higher lag of accommodation was observed in participants with lower total accommodative response in the reading and movie tasks (Pearson’s correlation: r = − 0.71, *P* < 0.0001, and r = − 0.64, *P* < 0.0001 for reading and movie tasks respectively; Fig. 2C,D). After controlling for refractive error, the lag of accommodation increased with age in the reading (r = 0.39, P = 0.0003) and movie (r = 0.21, P = 0.04) tasks. There were no significant associations observed between pupil size and accommodative lag across the two tasks: r = 0.10, *P* = 0.34 and r = − 0.11, *P* = 0.31, for the reading and movie tasks, respectively.

### Relationship between accommodative accuracy and stability, total accommodative response, and refractive error

In the time domain analysis (RMS), there was more instability in the accommodative response in the movie task (0.15 ± 0.05D) compared to the reading task (0.13 + 0.06D), (F_(1,180)_ = 4.45, *P* = 0.04; 2 × 2 mixed ANOVA analysis, dependent variable being root mean squared error of accommodation, and factors being task type (reading/movie) and refractive group (emmetropes/hyperopes)). There were no refractive group differences (F _(1, 180)_ = 1.09, *P* = 0.30), and no significant interactions observed between the two factors: F _(1, 192)_ = 0.33, *P* = 0.57. Similarly, in the frequency domain analysis (LFC), the accommodative response was more unstable in the movie task (1.2E−3 ± 7.71E−4) than reading task (7.65E−4 ± 7.42E−4), (F _(1,180)_ = 14.12, *P* = 0.0002; 2 × 2 mixed ANOVA analysis,) with no refractive group effect (*P* > 0.0.5 in both domains).

**Figure 3:**
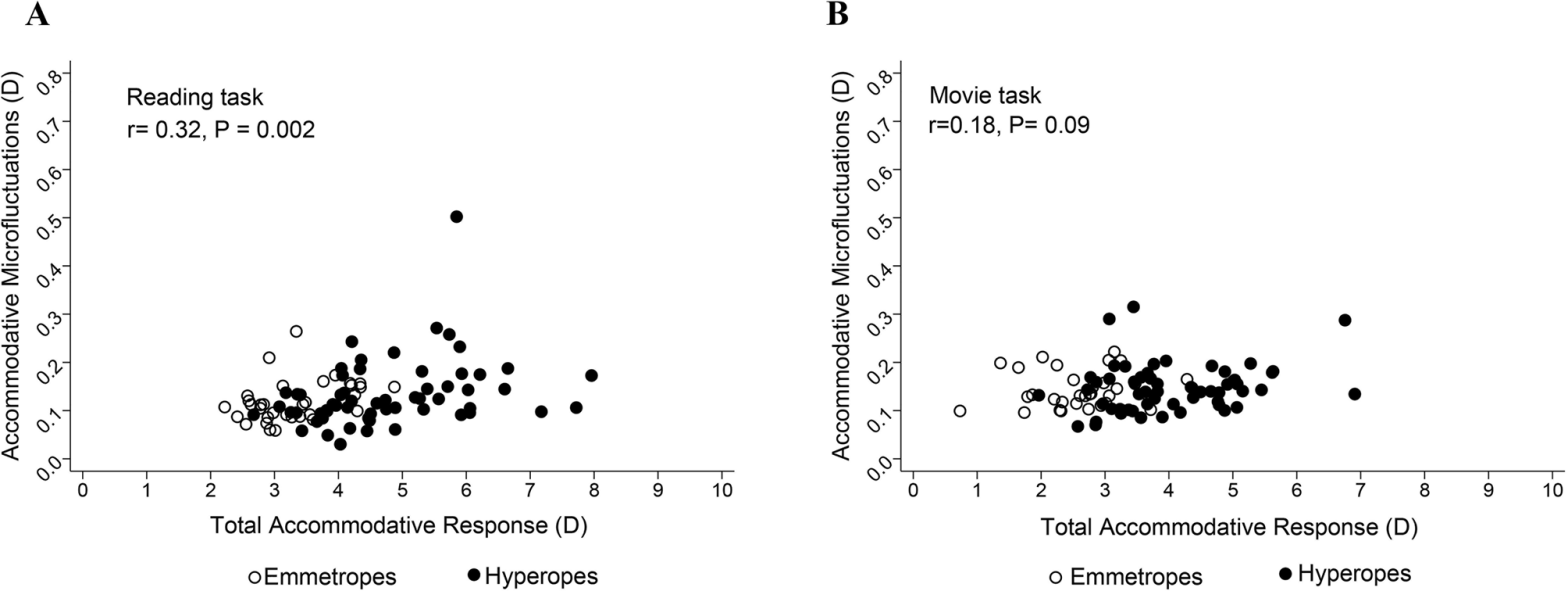
Scatter plots showing relationship between accommodative microfluctuations (RMS) and total accommodative response in the reading (**A**) and movie (**B**) tasks in all participants. Black hollow circles represent participants with emmetropia, while black solid circles represent participants with hyperopia.

Across the two tasks, the stability of the accommodative response was not related to the magnitude of the refractive error, in both time domain (RMS) and frequency domain (LFC) (*P* > 0.05 in both domains). However, the RMS of the accommodative microfluctuations increased with the total accommodative response in the reading task (r = 0.32, *P* = 0.002) but not the movie task (r = 0.18, *P* = 0.09), Fig. 3), and uncorrected hyperopes tended to have greater total accommodation across the two tasks (r = 0.78, *P* < 0.0001, and r = 0.75, *P* < 0.0001 for reading and movie tasks respectively). A multiple regression model was used to assess the relationship between the stability of the accommodative response (RMS) and the total accommodative response, after adjusting for pupil size, age across the two tasks (Table 2). Significant relationship between the stability of the accommodative response (RMS) and total accommodation was only observed in the movie task (*P* < 0.001) after controlling for pupil size and age.

**Table 2:** Multiple regression analysis of accommodative stability (time-domain, RMS) with independent variables age, pupil size, and total accommodative response in the reading and movie tasks.

Accommodative variability (D)		β coefficient	95% Confidence interval	P-value
Reading task	Age	− 0.013	− 0.019 to − 0.008	< 0.001
	Pupil size	− 0.030	− 0.043 to − 0.018	< 0.001
	Total accommodative response	0.005	− 0.002 to 0.012	0.160
	Intercept	0.364	0.274 to 0.454	< 0.001
Movie task	Age	0.003	− 0.005 to 0.006	0.920
	Pupil size	− 0.036	− 0.051 to − 0.021	< 0.001
	Total accommodative Response	0.010	0.002 to 0.017	< 0.001
	Intercept	0.303	0.197 to 0.408	< 0.001

No significant associations were observed between the LFC of accommodative microfluctuations and the magnitude of refractive error for the reading and movie tasks (*P* > 0.05 for both tasks).

### Associations between vergence response (accuracy and stability), refractive error, and accommodative response

Overall, the vergence response was higher in the reading task (2.91 ± 0.49 MA) compared to the movie task (2.67 ± 0.72 MA), and the observed difference was statistically significant (F_(1,179)_ = 5.51, *P* = 0.02; 2 × 2 mixed ANOVA analysis, dependent variable being vergence response, and factors being task type (reading/movie) and refractive group (emmetropes/hyperopes), with no group effect (*P* > 0.05)).

**Figure 4:**
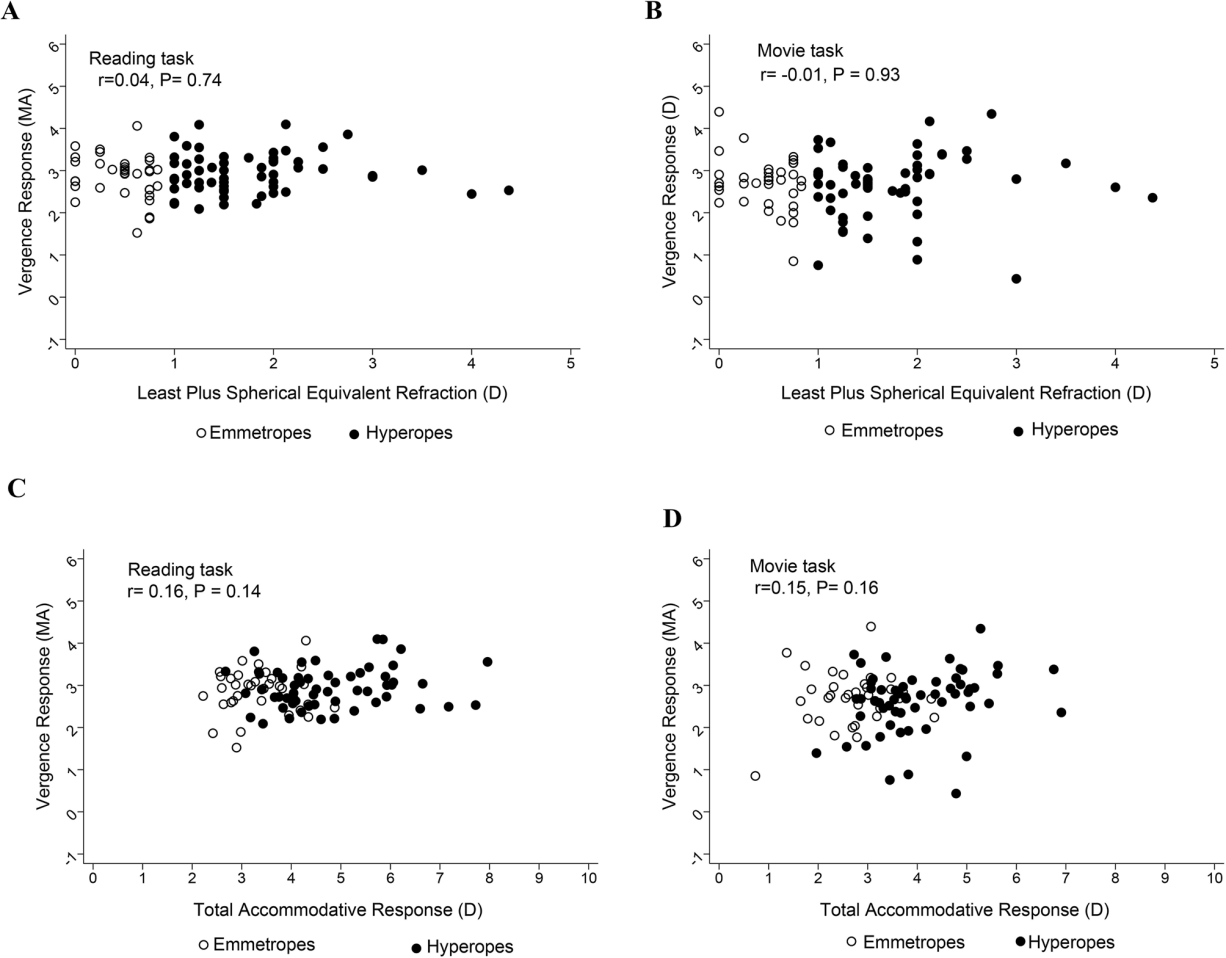
Scatter plots showing relationship between vergence response and refractive error in the reading (**A**) and movie (**B**) tasks. The relationship between vergence response and total accommodative response in the reading and movie tasks is shown in panels (**C** and **D**). Black hollow circles represent participants with emmetropia, while black solid circles represent participants with hyperopia.

There were no statistically significant associations between the magnitude of refractive error and mean vergence response across both tasks (Pearson correlation: r = 0.04, *P* = 0.74, r = − 0.01, *P* = 0.93 in reading and movie tasks respectively, Fig. 4A,B). Similarly, there were no significant associations between vergence response and the total accommodative response (r = 0.16, *P* = 0.14, and r = 0.15, *P* = 0.16 for reading and movie tasks respectively, Fig. 4C,D).

**Figure 5:**
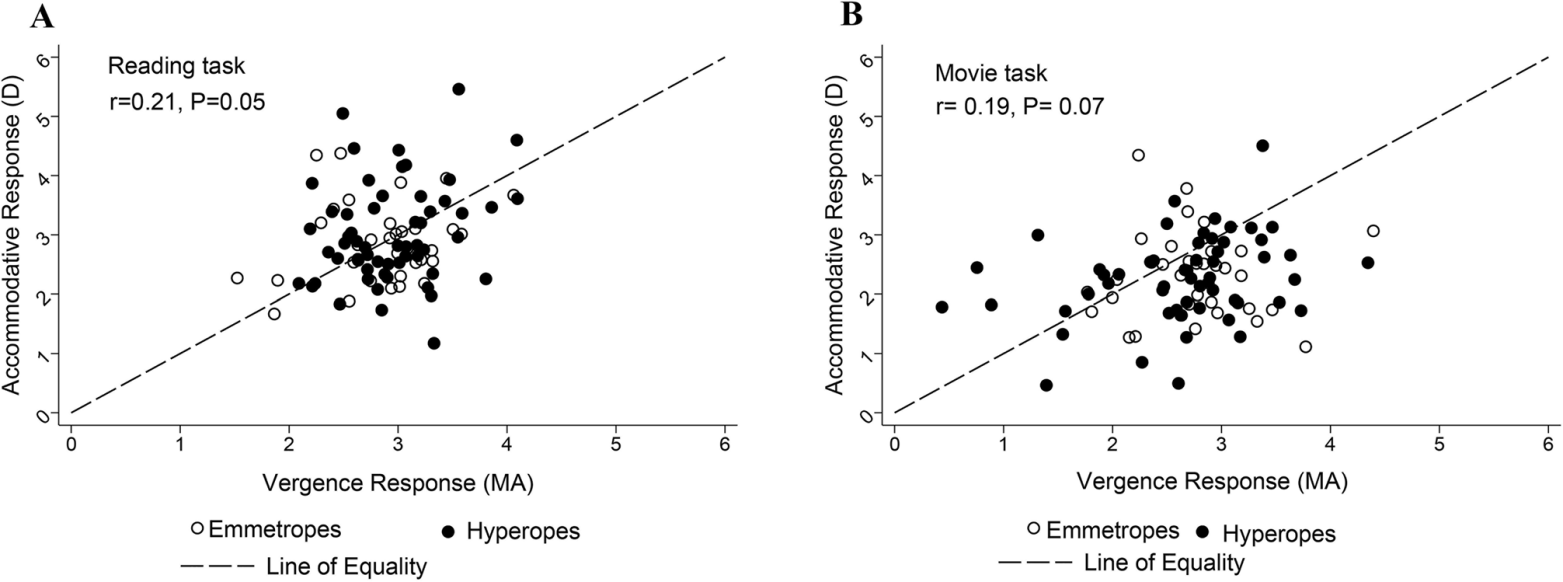
Scatter plots showing relationship between accommodative response and vergence response in the reading (**A**) and movie (**B**) tasks. Long dashed lines represent line of equality. Black hollow circles represent participants with emmetropia, while black solid circles represent participants with hyperopia.

The relationship between the mean vergence response and the mean accommodative response was not significant in both tasks (r = 0.21, *P* = 0.05 and r = 0.19, *P* = 0.07 for reading and movie tasks respectively), Fig. 5A,B.

The stability of the vergence response was considered using the standard deviation of the mean vergence response over the 15-min duration. There was more instability in the vergence response in the reading task (1.91 ± 0.19 MA) compared to the movie task (1.34 ± 0.17 MA), (F_(1,180)_ = 423.53, *P* < 0.0001; 2 × 2 mixed ANOVA analysis, dependent variable being vergence response, and factors being task type (reading/movie) and refractive group (emmetropes/hyperopes).

An investigation to determine whether the vergence instability increased with the mean vergence response in both tasks revealed no significant relationships (*P* > 0.05 in both tasks). Stability in the vergence response across the two tasks was not related to the stability of the accommodative response in both time and frequency domains (*P* > 0.05 in both domains). However, participants with greater total accommodation tended to have more unstable responses in their vergence response particularly in the movie task (r = 0.23, *P* = 0.03).

## Discussion

The present study assessed the characteristics of the accommodative and vergence responses in children with varying levels of natural, uncorrected hyperopia during two sustained near tasks, by comparing them to emmetropic controls. Children frequently engage in these tasks at school and in the home, and the tasks reflect “real-life” use of the vergence and accommodation systems. These tasks also offered different visual demands, one an active reading task, and the other a passive viewing (movie) task, to investigate the two systems.

The results of our study indicate that the average accuracy of the accommodative response over a 15-min time period to a 4D target demand was not related to the magnitude of uncorrected refractive error present. Children with uncorrected hyperopia demonstrated comparable accommodation to their emmetropic controls, although the magnitude of the response demonstrated in the reading and movie tasks by both groups was lower than expected for the 4D target demand. This finding is consistent with a previous study^7^, which examined accommodative performance in response to a 3D target demand across a range of uncorrected refractive errors (− 0.37 to + 4.58D) during a 10-min viewing period in children aged three to less than 10 years. Our study provides further evidence that uncorrected hyperopes exhibit sufficient average accommodation to achieve similar level of focusing to children with lower refractive errors. Moreover, in the present study, when the total accommodative response produced at 25 cm is considered, it becomes evident that the hyperope produces relatively more accommodation compared to their emmetropic counterparts. In the absence of amblyopia and strabismus, some hyperopes may be able to accommodate as accurately as other children^1^, but the stability of this response, and whether such prolonged accommodative demand is associated with oculomotor stress, including symptomatic presentation by patients, needs further investigation. There are a number of studies which have reported conflicting results on the accommodative response in uncorrected hyperopia, noting that significant accommodative lags are demonstrated by uncorrected hyperopes^1,4,5^. A number of factors may explain this conflict; the accommodative response in earlier reports was assessed for few seconds whereas in the present study and that of Roberts et al.^7^, a sustained accommodative response was studied. Moreover, in the work by Horwood and Ridell^1^, although a photorefraction technique was employed, five testing distances (25, 33, 50, 100 and 200 cm, with testing target positioned randomly) were explored and the target used was a large, high contrast, looming cartoon target. Candy et al.^2^ used Nott retinoscopy to measure accommodation responses in typically developing children whilst they viewed a high contrast cartoon picture positioned at 50 cm for a few seconds. In contrast, the present study employed two targets positioned at a 25 cm test distance both designed to simulate near activities typically engaged by children for both educational and recreational activities.

The characteristics of the vergence response, and the interaction between the vergence and accommodative motor systems in childhood hyperopia have not been extensively discussed in the literature. However, this topic is important given that the relationship between the two motor systems may help to understand which hyperopic cohorts are at risk of abnormal visual development such as strabismus^2^. In the present study, the accommodative responses of naturally uncorrected young hyperopes did not differ significantly from those of emmetropic controls, which consequently leads to the question of the relationship between vergence response and refractive error. The present study revealed that vergence responses were also independent of refractive error in either the movie or reading tasks. Participants with uncorrected hyperopia demonstrated vergence responses similar to emmetropic controls in both tasks—a finding in line with the comparable accuracy of their accommodative results. An association between the accommodative and vergence responses in both tasks was weak and of borderline significance. Nonetheless, a previous study in hyperopes found vergence responses to be accurate even when significant lag in accommodation was observed^1^. Our understanding of the dynamic relationship between the accommodation and vergence responses is based on the Schor’s model where cross-link interactions between the two motor systems occur mainly in the phasic (initial) stage of the response, and dissipate during the sustained, tonic component of the response^28^. Our study which investigated sustained accommodative and vergence responses likely tested the slow, sustained, tonic component of Schor’s model by which time no cross-link interactions were at play, with the consequent lack of significant relationships between the two motor systems.

While the parameters of this study did not examine eye tracking, it is likely that saccadic eye movements were somewhat different between the two tasks. Dorr et al.^29^ report a preference to maintain gaze near the centre of the screen while watching a video, while the nature of the reading task meant that the individual conducted small saccadic eye movements and fixations from left to right while reading text. It is possible that the increased saccadic eye movements during reading helped drive more accurate vergence eye movements. However, the different visual demands between the two tasks could also yield a notable potential difference in vergence and accommodative performance.

Results of the present study reveal increased accommodative instability in the passive task (movie) compared to the active task (reading) in both time and frequency domain analyses, in both refractive groups. Attentional differences between the active task (reading) and passive task (movie viewing) could partly explain the observed differences between the two tasks^7,8^. The reading task demanded more active participation as participants were required to read text aloud on a kindle/LCD monitor compared to the movie task. The increased task demand could be associated with the increased accommodative response observed, which also tended to be more stable, consistent with the outcomes described by Roberts et al.^7^ Although uncorrected hyperopes in the present study demonstrated more accommodative instability than the emmetropic controls across the two tasks, this finding did not reach statistical significance. However, given that uncorrected hyperopes tended to have greater total accommodative response, which was associated with higher RMS of accommodative microfluctuations, our finding still confirms the reported association between unstable accommodative response and uncorrected hyperopia^7,30^. Our study used low to moderate uncorrected hyperopes, and a repetition of this experiment in those with higher levels of uncorrected hyperopia may reveal that at higher magnitudes, those with hyperopia may not be able to maintain sufficient accuracy and stability for clear and comfortable reading. Definitive functional roles of accommodative microfluctuations are yet to be fully elucidated, however, it has been suggested that they may be a mechanism to provide feedback error to maintain appropriate response during steady-state accommodation^24,27,31^. It has also been reported that where there is increased accommodative effort, such as may occur in higher magnitude of hyperopia, there is a decreased zonular tension which causes the lens to move freely resulting in increased microfluctuations^32–34^. Although microfluctuations may have functional roles, they also represent temporal instability in the retinal image quality^24,35^, and have the potential to cause visual discomfort^36,37^, which may present in some hyperopes as asthenopia. Such asthenopic symptoms could negatively impact the ability of uncorrected hyperopes to undertake sustained and effective engagement with near tasks commonly associated with schoolwork and become detrimental to their academic performance^7,38^. Previous studies suggest that small pupil sizes, and young age are factors which affect accommodative microfluctuations^31,39^. However, in the present study of similarly aged children, only pupil size was consistently related to accommodative microfluctuations across the two tasks.

There was inter-task difference in the stability of the vergence response, with increased vergence instabilities in the reading task compared to the movie task. Again, the reading task being the more visually demanding, perhaps, elicited more unstable responses. There is a paucity of data describing the relationship between the stability of the vergence response and refractive error, and the clinical relevance of such data. Across the two tasks, instability in the vergence response was higher in uncorrected hyperopes than emmetropes (Table 1). Although no functional role or clinical significance has previously been reported for such instabilities in the vergence response, one may speculate that they could play a role in the asthenopic symptoms experienced by some hyperopes when they undertake sustained near activities, given that instability in vergence response increased with accommodative demand (total accommodative response) in this study.

Our study provides insights into sustained vergence characteristics of uncorrected hyperopes under two visual demands to complement our understanding of the two oculomotor systems under closed-loop conditions. The present study was designed to elicit accommodation and vergence during the slower, tonic response, rather than to address the magnitude of cross-link interactions between accommodation and vergence which occur during the fast, phasic response^28^. Further studies are required to advance our understanding of the cross-link interactions between accommodation and vergence in hyperopia. Other limitations include that our maximum level of hyperopia was + 4.38D mean spherical equivalent, and it would be valuable to include more moderate to high levels of hyperopia in future. In common with other studies of accommodation using photorefraction, it is not possible to determine true underlying defocus, but the use of relative measures (i.e. a change in defocus), individual calibration, and scrutiny of group level patterns, overcomes the impact of potential inaccuracies for individual participants. Strengths of this study include a large sample size, and a long time frame (15 min) to evaluate accommodative and vergence responses in both reading and movie tasks.

## Conclusion

This study revealed that the average magnitude of accommodative and vergence responses demonstrated during sustained near vision tasks are comparable between natural uncorrected hyperopes and emmetropes. Furthermore, accommodative and vergence instabilities increased in uncorrected hyperopes who demonstrated greater accommodative responses, which may have implications for the visual experience of the uncorrected hyperope during sustained near vision activity.

## Data Availability

The datasets generated during and/or analysed during the current study are available from the corresponding author on reasonable request.
